# Genome-Wide Identification and Characterization of the BZR Transcription Factor Gene Family in *Leymus chinensis*

**DOI:** 10.3390/genes16020155

**Published:** 2025-01-26

**Authors:** Ruiqi Zhao, Jiayuan Cheng, Yingjie Yu

**Affiliations:** College of Life Sciences, Jilin Normal University, Siping 136000, China; zhaoruiqi0719@163.com (R.Z.); jiayuan04102024@163.com (J.C.)

**Keywords:** BZR family, BR signaling pathway, *Leymus chinensis*, plant growth

## Abstract

Background/Objectives: The BZR gene family, a critical transcription factor in the brassinosteroid (BR) signaling pathway, regulates plant growth and development. Despite its significance, the BZR gene family in *Leymus chinensis*, a valuable forage grass renowned for its stress tolerance and nutritional quality, remains uncharacterized, and its functional roles are largely unexplored. Methods: Employing advanced bioinformatics tools, we conducted a genome-wide survey to identify members of the BZR gene family in *L. chinensis*. Phylogenetic analyses were performed to classify these genes into distinct clades, while gene structure and conserved motif analyses assessed their evolutionary conservation and potential regulatory mechanisms. Additionally, transcriptome sequencing was utilized to examine the expression patterns of BZR genes in response to simulated animal grazing. Results: Eight *LcBZR* genes were identified, evenly distributed across all seven chromosomes. Phylogenetic analysis categorized these genes into three distinct groups, reflecting their evolutionary relationships. Most *LcBZR* genes exhibited highly conserved gene structures and motifs, with promoters enriched in *cis*-acting elements such as G-box and ARE. Expression profiling revealed that *LcBZR* genes are predominantly expressed in key tissues, particularly leaves and roots, suggesting their involvement in critical physiological processes. Transcriptomic analysis demonstrated that simulated animal grazing modulated the expression levels of *LcBZR* genes, implicating their role in promoting cellular elongation and division through the BR signaling pathway. Conclusions: This study highlights the crucial role of *LcBZR* genes in regulating plant growth, development, and response to environmental stimuli, providing a foundational basis for understanding the molecular mechanisms of BR-mediated plant development and stress adaptation.

## 1. Introduction

The growth and development of plants is a complex process influenced by a dynamic interplay of environmental factors, internal signals, and genetic information, which collectively shape plant morphology [1]. Plant hormones such as auxins, cytokinins (CTKs), gibberellins (GAs), abscisic acid (ABA), brassinosteroids (BRs), and strigolactones (SLs) play fundamental regulatory roles in plant growth and development. Among these extensively studied hormones, BRs and SLs have particularly captured our interest. Despite their relatively low concentrations within plants, they exert significant physiological effects. Brassinosteroids (BRs) are a class of plant hormones characterized by their polyhydroxylated sterol structure [2]. Multiple studies have demonstrated their functions in mediating root growth and development, regulating cell elongation and division, and enabling plants to adapt to environmental challenges, including abiotic stresses [3,4,5]. SLs are crucial in maintaining plant structure and significantly mitigate the adverse effects of external stresses such as drought, nutrient deficiency, heavy metals, and salinity through their low concentration and high efficiency [6]. SLs not only function similarly to BRs, but also act synergistically with BRs in certain processes, jointly influencing plant growth. Studies have shown that BRs and SLs exhibit a synergistic effect in alleviating salt stress in maize [7]. Further research has revealed that in tomato, signaling molecules such as SLs and carbohydrates may regulate BR synthesis and signal transduction through cytokinin-dependent or -independent pathways, thereby affecting bud branching growth [8]. Therefore, the regulatory mechanisms of BR signaling warrant in-depth investigation.

Over the past two decades, the BR signaling pathway has been extensively studied, revealing detailed regulatory mechanisms [4]. The initiation of BR signaling involves the BAK1/BRI1 receptor complex, composed of brassinosteroid insensitive 1 (BRI1), a leucine-rich repeat (LRR) receptor-like kinase, and BRI1-associated receptor kinase 1 (BAK1). These two components work in synergy to perceive and transduce BR signals [9,10]. The BR signaling pathway exhibits a sophisticated regulatory network, encompassing both positive and negative regulatory mechanisms. Positive regulation primarily operates through the activation of the transcription factors BES1/BZR1, which promote the expression of BR-responsive genes, thereby enhancing BR signaling [9,11]. Conversely, negative regulation involves inhibitory elements such as the kinase BIN2, which phosphorylates BES1/BZR1, leading to their degradation and attenuating BR signaling, thus adversely affecting plant growth and development [12].

The BZR gene family was initially identified in *Arabidopsis thaliana*, where it has since been extensively studied [9]. This study experimentally strongly supports a high degree of similarity between BES1, BZR1, and other predicted *Arabidopsis* proteases (including BEH1-4), suggesting that they may perform partially overlapping functions in BR signaling pathways. In the BR signaling pathway and anther development of *Arabidopsis*, BZR transcription factors (members of the BZR1/BES1 family) play a critical role. Notably, studies have demonstrated that BZR transcription factors can regulate anther chamber development through a mechanism independent of BR receptors [13]. Chung et al. explored the interplay between abscisic acid (ABA) and BRs, revealing that BIN2 not only participates in ABA-mediated salt tolerance, but also mediates the synergistic regulation of target gene expression by ABA and BRs, enabling the precise control of *Arabidopsis* growth under environmental stress [14]. Furthermore, it has been shown that BZR1 positively regulates plant frost tolerance through both CBF-dependent and CBF-independent pathways [15].

However, the specific functions of the BZR gene family in other plant species remain poorly understood, with studies limited to a handful of species. For instance, a genome-wide analysis of the BZR gene family in bread wheat identified 20 *TaBZR* genes, which were analyzed in detail to elucidate their structural and functional characteristics [16]. Similarly, recent experiments identified eight BZR genes in potatoes, with some genes exhibiting high expression levels, indicating their involvement in both plant hormone signaling and stress response pathways, enabling plants to better adapt to environmental changes and maintain normal growth and development [17]. In sugar beet, an analysis of *BvBZR* genes revealed expression in roots, stems, and leaves, with significantly higher expression levels in leaves compared to other tissues [18]. The BZR gene family also plays crucial roles in regulating responses to biotic and abiotic stresses. For example, in legumes, analyses of intron–exon structures and expression quantification demonstrated that BZR genes significantly regulate organ development and exhibit remarkable responses to drought and salt stresses [19]. In cucumber, six members of the *CsBZR* gene family were identified, with a qRT-PCR analysis showing that *CsBZR* genes respond to hormones and abiotic stresses [20]. Similarly, *NbBZR* genes in *Nicotiana benthamiana* were found to respond to cold, heat, and salt stresses, highlighting their multifunctional roles in stress resistance [21].

*Leymus chinensis* (*Trin.*) *Tzvel.*, a perennial grass species, is widely distributed across the grasslands of Eurasia. Due to its superior forage quality and stress resistance, *L. chinensis* holds significant importance in animal husbandry and ecological conservation, making it a valuable forage grass [22]. However, recent changes in ecological conditions and human-induced disturbances have severely threatened the genetic diversity and adaptability of *L. chinensis*. Additionally, its remarkable tolerance to salt, alkali, frost, and drought stresses has garnered considerable attention [23,24]. Despite the wealth of research on BZR genes in other plants, little is known about their roles in *L. chinensis*. To address this gap, we employed bioinformatics approaches to identify and analyze BZR transcription factors in *L. chinensis*. This included examining genomic localization, protein motif structure, phylogenetic relationships, structural features, and the chromosomal localization of BZR genes. Our findings provide fundamental insights into the composition and specific expression of BZR genes in *L. chinensis*. This study lays a solid foundation for future research on the roles of BZR genes in growth, development, and responses to abiotic stresses in this vital grass species.

## 2. Materials and Methods

### 2.1. Identification of BZR Gene Family Members in L. chinensis

To identify the BZR gene family in *L. chinensis*, protein sequences of six BZR genes from *A. thaliana* were downloaded from the TAIR database (https://www.arabidopsis.org/, accessed on 15 November 2024). Similarly, four BZR protein sequences from rice were retrieved based on previously published data [10]. Additionally, the conserved BZR domain (PF05687) was obtained through InterPro (https://www.ebi.ac.uk/interpro/, accessed on 15 November 2024) using one BZR sequence from *A. thaliana*. The collected sequences were used for comparative analysis against the *L. chinensis* genome. Results from the three methods were filtered and cross-validated using NCBI tools, and the union of all candidates was taken to determine the final set of *LcBZR* genes.

### 2.2. Physicochemical Properties and Subcellular Localization

The physicochemical properties, such as amino acid length, molecular weight, and isoelectric point, of the identified *LcBZR* proteins were analyzed using TBtools software (Version 2.152). Subcellular localization was predicted using the Cell-PLoc 2.0 server (http://www.csbio.sjtu.edu.cn/bioinf/Cell-PLoc-2/, accessed on 15 November 2024) [25].

### 2.3. Chromosomal Localization

The chromosomal distribution of *LcBZR* genes was visualized using TBtools software (Version 2.152) based on the GFF3 annotation file of *L. chinensis* [25]. A chromosome localization map was generated to display the precise positions of the *LcBZR* genes.

### 2.4. Phylogenetic, Synteny, and Selection Pressure Analysis

A phylogenetic tree was constructed using BZR gene sequences from *L. chinensis*, *A. thaliana*, *Oryza sativa*, *Ginkgo biloba*, and *Physcomitrella patens* with MEGA software (Version 11.0.13), followed by visualization and refinement using EvolView [26]. A synteny analysis was performed with McScanX to identify homologous gene pairs. TBtools was then used to visualize the synteny relationships between *L. chinensis* and *A. thaliana*, as well as *L. chinensis* and *O. sativa*. A selection pressure analysis within *LcBZR* gene pairs was conducted based on synonymous (Ks) and nonsynonymous (Ka) substitution rates [27].

### 2.5. Gene Structure, Conserved Motif, and Functional Domain Analysis

Conserved motifs of *LcBZR* proteins were identified using the MEME Suite (https://meme-suite.org/, accessed on 22 November 2024) with the maximum motif parameter set to 10 and other parameters set to default [28]. The identified motifs, together with gene structures, were visualized using TBtools. A functional domain analysis was performed via NCBI Batch CD-Search (https://www.ncbi.nlm.nih.gov/Structure/bwrpsb/bwrpsb.cgi, accessed on 22 November 2024) and visualized using TBtools software (Version 2.152).

### 2.6. Cis-Acting Regulatory Element Analysis of LcBZR Promoters

Promoter sequences of *LcBZR* genes were extracted using TBtools software (Version 2.152) and analyzed for *cis*-acting regulatory elements using the PlantCARE database (https://bioinformatics.psb.ugent.be/webtools/plantcare/html/, accessed on 25 November 2024) [29]. Relevant elements were identified, filtered, and categorized for further analysis.

### 2.7. Expression Analysis of LcBZR Genes in Different Tissues

Expression data for *LcBZR* genes were retrieved from the National Genomics Data Center (NGDC, https://ngdc.cncb.ac.cn/gsa/browse/CRA011402, accessed on 28 November 2024). The data were processed, and a heatmap was generated using TBtools to visualize gene expression levels across various tissues, with color scales representing relative expression values [25].

### 2.8. L. chinensis Plants and the Mimicked Grazing Treatment

*L. chinensis* seedlings were selected and potted under greenhouse conditions one month after natural regreening. The pots, with a 20 cm diameter and 15.5 cm depth, were filled with a 1:2 mixture of vermiculite and nutrient soil to a depth of 14 cm. The greenhouse was maintained at 25 °C, 70% humidity, with a 16 h light/8 h dark cycle. Once the seedlings had grown five or six new leaves, they were randomly assigned to two groups: CK (control) and S (saliva-treated). The control group remained uncut, while a quarter of the aboveground parts were excised in the S group. Cow saliva was collected by allowing cows to chew on a sponge wrapped around chopsticks after being fed fresh plants. The collected saliva, which mimicked that left on plants during grazing, was stored on ice and processed within 2–3 h. All instruments were disinfected with 75% alcohol, and no animals were harmed during the collection process. After treatment, plant samples were taken at various time points, quickly frozen in liquid nitrogen, and stored at −80 °C for further analysis. At least three biological replicates were used for all experiments. Further processing details can be found in another study [30].

## 3. Results

### 3.1. Chromosomal Distribution of the BZR Gene Family in L. chinensis

**Figure 1:**
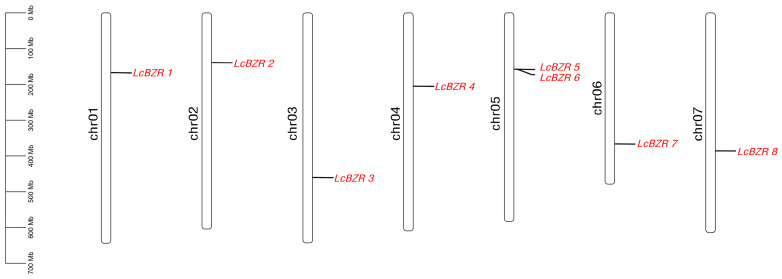
Distribution of the *LcBZR* genes on chromosomes. The left scale indicates chromosome length.

Across the entire genome of *L. chinensis*, we successfully identified eight BZR genes. Based on the gene annotation information of *L. chinensis*, we utilized TBtools software to visually map the specific loci of these BZR genes on chromosomes. The results revealed that these eight BZR genes are precisely located on seven distinct chromosomes (Table S1). We designated these genes as *LcBZR1*, *LcBZR2*, *LcBZR3*, *LcBZR4*, *LcBZR5*, *LcBZR6*, *LcBZR7*, and *LcBZR8*, according to their chromosomal positions. Specifically, one BZR gene was identified on each of the chr01, chr02, chr03, chr04, chr06, and chr07, while two BZR genes were localized on the chr05, forming a gene cluster (Figure 1). These findings provide an important foundation for further analysis of the functional roles of BZR genes in *L. chinensis*.

### 3.2. Identification and Physicochemical Properties of the BZR Gene Family in L. chinensis

Using species comparison and conserved sequence identification methods, we characterized the BZR gene family in *L. chinensis*, identifying a total of eight members (*LcBZR1–LcBZR8*). The lengths of these genes ranged from 119 to 387 amino acids, with the majority of members spanning 300 to 400 amino acids. Correspondingly, their molecular weights varied from 14 kDa to 41 kDa, and their isoelectric points ranged from 4.88 to 10.18. A subcellular localization analysis indicated that all BZR gene family members in *L. chinensis* are predominantly located in the nucleus (Table 1). These results provide valuable insights into the structural and functional characteristics of BZR genes in *L. chinensis*, suggesting their potential roles in nuclear processes related to gene regulation.

### 3.3. Phylogenetic Analysis of LcBZR Genes

**Figure 2:**
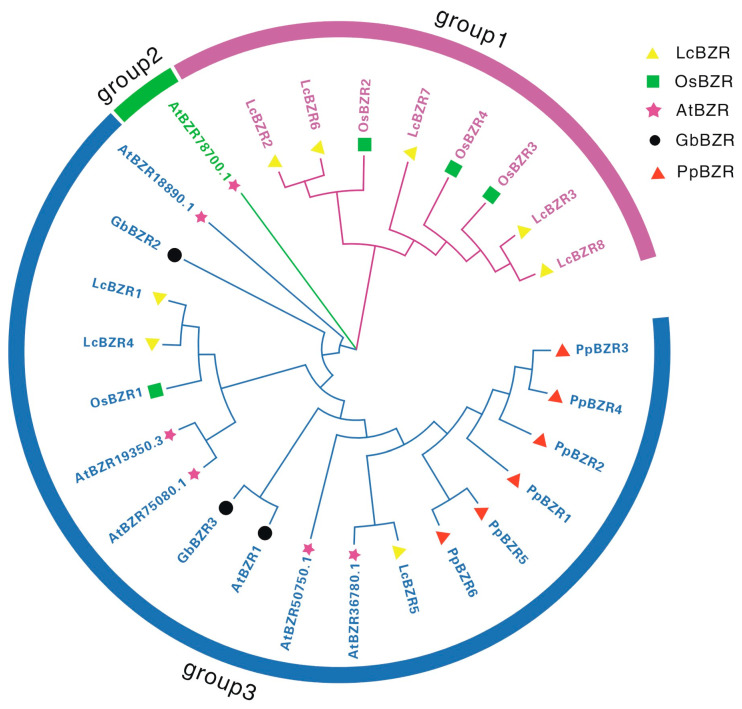
Phylogenetic analysis of the *LcBZR* gene family. The phylogenetic tree, constructed using sequences from *L. chinensis* (Lc), *A. thaliana* (At), *O. sativa* (Os), *G. biloba* (Gb), and *P. patens* (Pp), is divided into three groups, with each group represented by a distinct color.

To explore the evolutionary relationships of the BZR gene family across diverse species, including *L*. *chinensis*, *A*. *thaliana*, *O*. *sativa*, *G*. *biloba*, and *P*. *patens*, we constructed a phylogenetic tree using eight *LcBZR* protein sequences along with corresponding sequences from these species (Figure 2). The phylogenetic tree classified the BZR genes into three major groups: Group I contains eight BZR proteins, Group II consists of a single BZR protein, and Group III includes 18 BZR proteins. The results revealed that most *LcBZR* genes clustered closely with *O. sativa* BZR genes within the same evolutionary branch, suggesting a close phylogenetic relationship between *L. chinensis* and *O. sativa*.

### 3.4. Gene Structure and Conserved Motif Analysis of LcBZR Genes

**Figure 3:**
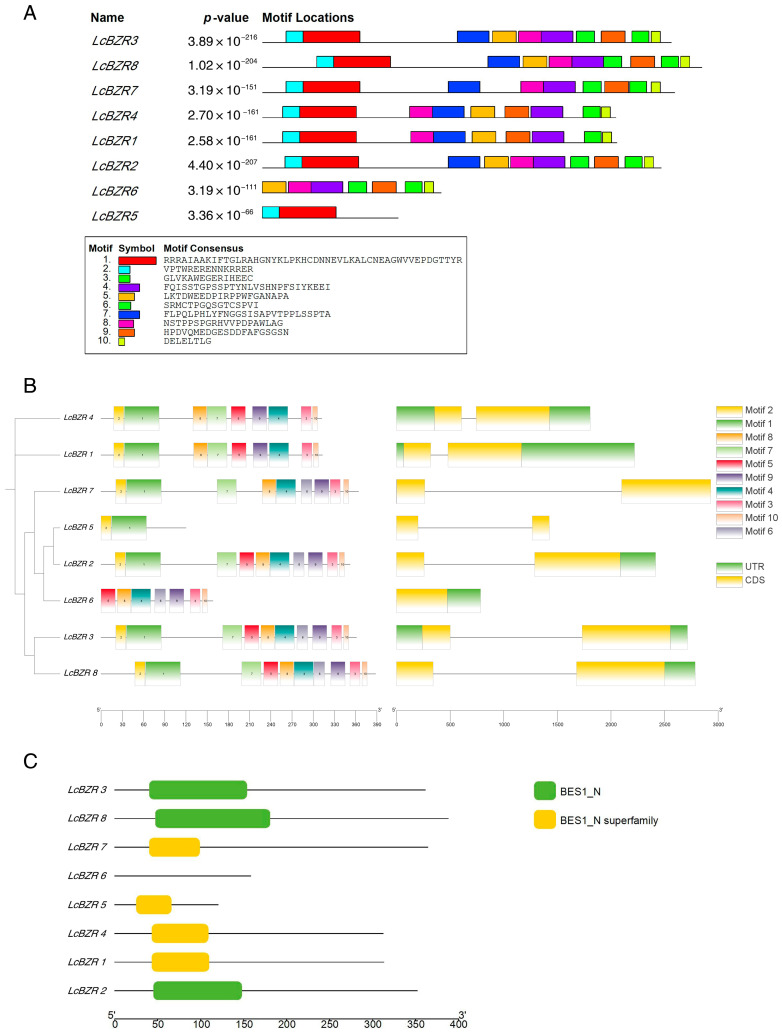
Structure and conserved motif visualization of the BZR gene family in *L. chinensis*. (**A**) Conserved motifs of *LcBZR* genes elucidated by MEME. Distinct conserved motifs are represented by different colored blocks. (**B**) The gene structure of *LcBZR* members. Green regions indicate untranslated regions (UTRs), yellow regions represent coding sequences (CDSs), and lines denote introns. (**C**) Conserved domain diagram of the *LcBZR* gene. Conserved BES1_N domains in *LcBZR* genes.

To investigate the structural characteristics of *LcBZR* genes, we utilized the MEME online tool to identify conserved motifs among the eight *LcBZR* family members. A total of ten conserved motifs were predicted (Table S2). As shown in Figure 3A, most *LcBZR* family members contain nine to ten motifs. However, *LcBZR5* contains only two motifs, and *LcBZR6* contains seven motifs, marking them as notable exceptions. A gene structure analysis revealed that *LcBZR6* lacks introns, whereas all other members possess a single intron (Figure 3B). Furthermore, all *LcBZR* genes were found to contain the BES1_N conserved domain, a hallmark feature of the BZR gene family (Figure 3C).

### 3.5. Cis-Acting Regulatory Element Analysis of LcBZR Genes

**Figure 4:**
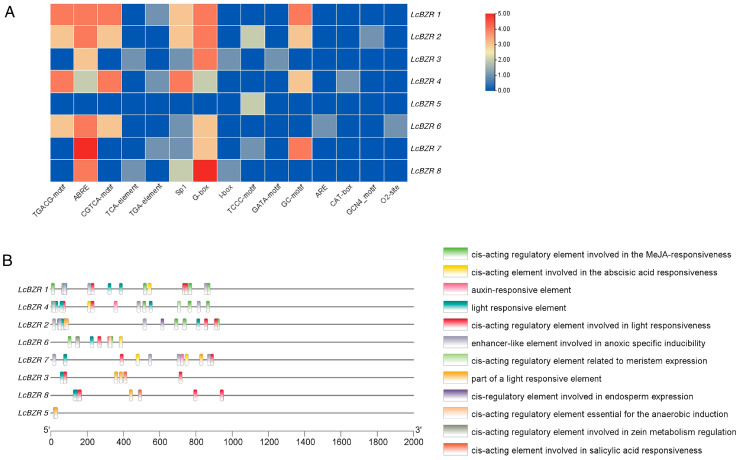
Predicted *cis*-acting regulatory elements in the promoter regions of *LcBZR* genes. (**A**) Number of *cis*-acting elements predicted in each *LcBZR* gene promoter region. (**B**) Positions of *cis*-acting elements within the promoter regions.

To gain deeper insights into the regulatory mechanisms of the *LcBZR* gene family, we performed a comprehensive prediction of *cis*-acting regulatory elements. Using the PlantCARE online server, we analyzed the 2000-bp upstream sequences of the translation start sites for each *LcBZR* gene. A total of 125 *cis*-acting elements, categorized into 15 types, were identified, including 59 hormone-responsive elements, 48 light-responsive elements, 15 stress-responsive elements, and 3 elements related to growth and development (Table S3). A visualization of these *cis*-acting elements revealed that the ABRE elements (associated with abscisic acid response) and G-box elements (involved in light response) were the most abundant (Figure 4A). Further analysis of the promoter regions indicated a significant enrichment of hormone-responsive elements, including those for methyl jasmonate, abscisic acid, and auxin, as well as light-responsive elements (Figure 4B). These findings suggest that the *LcBZR* gene family is extensively involved in various plant hormone signaling pathways and plays an important role in regulating plant growth and development.

### 3.6. Synteny Analysis of BZR Genes in L. chinensis

**Figure 5:**
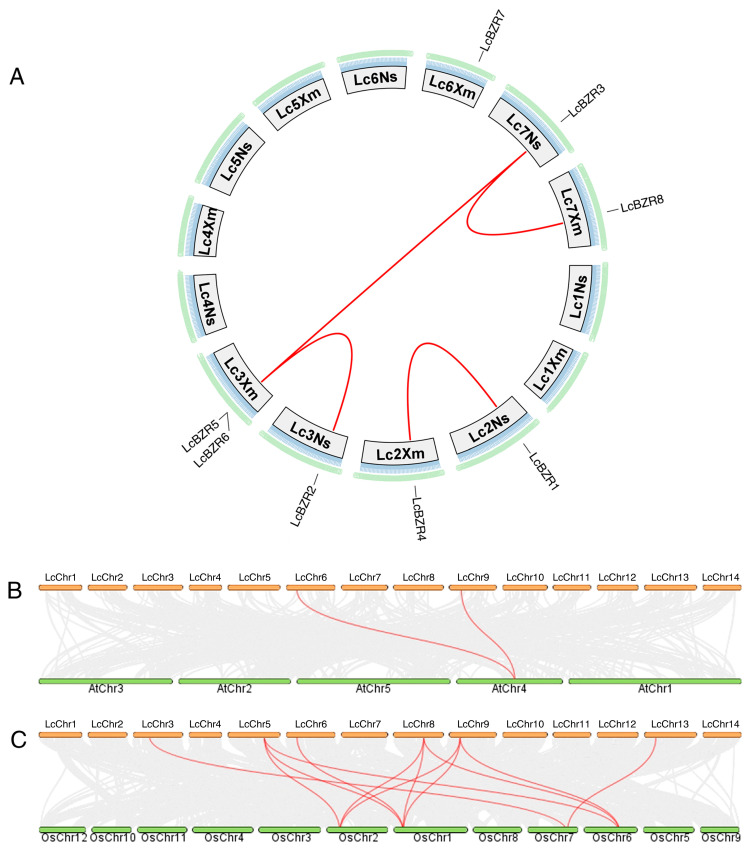
Synteny analysis of BZR genes in *L. chinensis* and related species. (**A**) Synteny relationships among BZR genes within *L. chinensis*. (**B**) Synteny relationships between *L. chinensis* and *A. thaliana*. (**C**) Synteny relationships between *L. chinensis* and *O. sativa*. Red lines highlight syntenic BZR gene pairs.

To further explore the evolutionary relationships of the BZR gene family between *L. chinensis* and other species, a synteny analysis was conducted using an online analysis tool. The results revealed four syntenic gene pairs within the BZR genes of *L. chinensis* (Figure 5A). Additionally, 12 syntenic gene pairs were identified between *L. chinensis* and *O. sativa*, and two syntenic gene pairs between *L. chinensis* and *A. thaliana* (Figure 5B,C). These findings suggest that the BZR gene family in *L. chinensis* shares a closer evolutionary relationship with *O. sativa* than with *A. thaliana*, indicating a greater similarity in genomic structure and a more recent common ancestor between *L. chinensis* and *O. sativa*.

### 3.7. Evolutionary Selection Pressure Analysis of LcBZR Genes

To explore the evolutionary selection pressures acting on *LcBZR* genes, we calculated the nonsynonymous substitution rate (Ka), synonymous substitution rate (Ks), and Ka/Ks ratio for four pairs of *LcBZR* genes (Table 2). A Ka/Ks ratio of 1 indicates neutral selection, a ratio between 0 and 1 (0 < Ka/Ks < 1) suggests purifying selection, and a ratio greater than 1 (Ka/Ks > 1) reflects positive selection. The Ka/Ks values for all four gene pairs ranged between 0 and 1, indicating that *LcBZR* genes have undergone purifying selection, highlighting their evolutionary conservation.

### 3.8. Tissue-Specific Expression Patterns of LcBZR Genes

**Figure 6:**
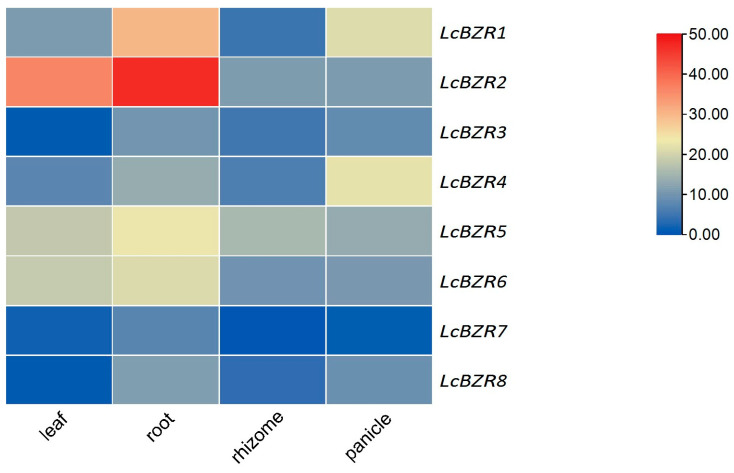
The heatmap illustrates the expression levels of *LcBZR* genes in four tissues of *L. chinensis*: roots, stems, leaves, and inflorescences.

To visualize the *LcBZR* gene expression in *L. chinensis*, we systematically analyzed the data and generated the expression heatmap with TBtools software. As shown in Figure 6, the expression levels of *LcBZR* genes were comprehensively analyzed across four tissues: roots, stems, leaves, and inflorescences. The results revealed that *LcBZR2* displayed significantly higher expression levels in the roots and leaves of *L. chinensis*, while *LcBZR7* exhibited consistently low expression across all four tissues. On a broader scale, the *LcBZR* genes were predominantly expressed in the root and leaf tissues, suggesting their essential roles in supporting growth and development in *L. chinensis*. In conclusion, the findings highlight that the *LcBZR* genes exhibit distinct expression patterns across different tissues, underscoring their functional differentiation in various physiological and developmental processes in *L. chinensis*.

### 3.9. Changes in BZR Genes Within the BR Hormone Signal Transduction Pathway Induced by Animal Grazing

**Figure 7:**
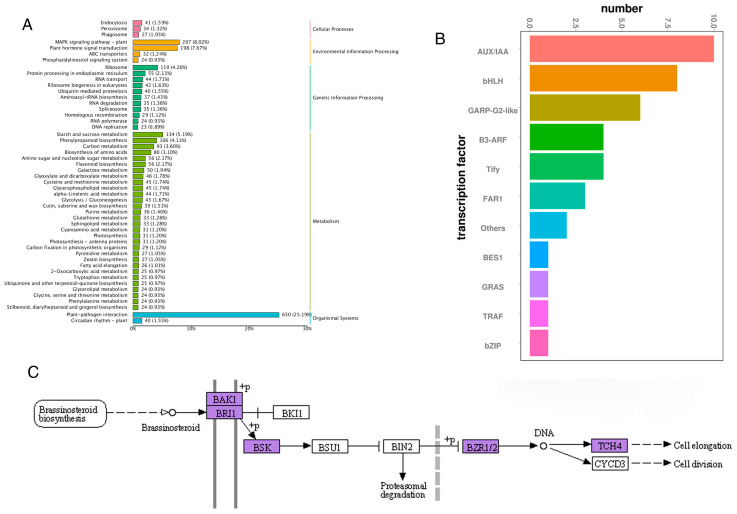
Differentially expressed gene (DEG) analysis of *L. chinensis* in response to simulated animal grazing. (**A**) KEGG pathway annotation of DEGs. (**B**) Transcription factor prediction for the 198 DEGs related to plant hormone signal transduction. (**C**) Gene expression changes in the BR hormone signal transduction pathway following simulated grazing treatment.

To investigate the effects of animal grazing on the BZR gene family within the brassinosteroid (BR) hormone signaling pathway in *L. chinensis*, this study employed a combination of cutting and the application of animal oral secretions to simulate grazing. Leaf tissues from both control groups (CK) and treatment groups (S) were collected at 2, 6, and 24 h post-treatment for transcriptome sequencing analysis. The analysis identified 198 differentially expressed genes enriched in the plant hormone signal transduction pathway through KEGG classification annotation following the simulated grazing treatment (Figure 7A). Further prediction of transcription factors within this pathway revealed differential expression of BES1 (Figure 7B). BES1 and BZR1 are pivotal members of the BZR gene family that serve as key transcription factors in the BR hormone signaling pathway. BES1 binds to *cis*-elements such as E-boxes in promoters and, in conjunction with BZR1, regulates the expression of downstream genes within the BR pathway. To elucidate the specific changes in BR signaling-related genes in response to simulated grazing, we conducted additional KEGG pathway analysis on the 198 differentially expressed genes. This analysis demonstrated that numerous gene families within the BR hormone signaling pathway exhibited significant alterations, indicating that grazing treatment elicited a response in the BR signaling pathway (Figure 7C). Within this metabolic pathway, the activated BRI1-BAK1 complex phosphorylates downstream kinases (e.g., BSK), initiating a cascade of phosphorylation and dephosphorylation events that transmit the signal to downstream components BSU1 and BIN2. This ultimately leads to the activation of transcription factors BZR1/2, which regulate cell division and elongation. These results indicate that animal grazing influences the growth and development of *L. chinensis* by modulating gene expression within the BR signaling pathway.

## 4. Discussion

The BZR gene family holds significant importance within the plant kingdom. As a vital group of transcription factors (TFs), BZR proteins not only constitute central components of the brassinosteroid (BR) signaling pathway, but also participate in a wide range of physiological processes [31]. Substantial evidence has revealed the multifaceted roles of the BZR gene family, which not only regulate plant growth and development, but also actively contribute to defenses against abiotic stresses [32,33]. However, before our study, no comprehensive investigation of the BZR transcription factor (TF) family in *L. chinensis* had been conducted.

Through a detailed genome-wide analysis, we identified eight members of the BZR gene family in *L. chinensis*. Comparatively, the number of BZR genes identified in other plant species varies, such as six in *A. thaliana*, seven in foxtail millet, eight in potato, six in cucumber, and six in sugar beet [10,17,18,20,34]. The number of BZR genes identified in *L. chinensis* aligns closely with these species, underscoring the high conservation of the BZR gene family throughout plant evolution. Further studies have reported uneven chromosomal distributions of BZR genes in some plants, such as potato, whereas in others, such as *Arabidopsis*, these genes are evenly distributed [17,34]. In this study, the eight BZR genes in *L. chinensis* were distributed evenly across its seven chromosomes, reflecting a pattern consistent with that of most plants. This even distribution may indicate functional diversification or redundancy, which warrants further investigation. Our findings provide a valuable foundation for understanding the functional roles of BZR genes in *L. chinensis*, particularly in stress response pathways and developmental processes. These results contribute to the broader understanding of the evolutionary conservation and functional divergence of the BZR gene family across plant species.

To better understand the evolutionary relationships within the BZR gene family, a phylogenetic tree was constructed using sequences from *L. chinensis*, *A. thaliana*, *O. sativa*, *G. biloba*, and *P. patens* (Figure 2). The *LcBZR* genes are well-integrated into the known BZR subgroups of *A. thaliana*, *G. biloba*, *P. patens*, and *O. sativa*, suggesting that *LcBZR* genes share a common evolutionary origin. Additionally, Group 3 contains a relatively larger number of members, suggesting that these *LcBZR* genes underwent duplication events during evolution. Interestingly, Group 1 is composed exclusively of monocot species, indicating that these genes may have originated in monocots and evolved specific functions. These unique functions might contribute to physiological adaptations and morphological traits, such as influencing leaf structure and specialized root development [35]. A synteny analysis further revealed that *LcBZR* genes exhibit some syntenic relationships within *L. chinensis* itself, but lack synteny with *GbBZR* and *PbBZR* genes (Figure 5). Two syntenic gene pairs were identified between *L. chinensis* and *A. thaliana*, suggesting a shared evolutionary history. In contrast, 12 syntenic gene pairs were observed between *LcBZR* and *OsBZR* genes, highlighting a closer evolutionary and genetic relationship between *L. chinensis* and *O. sativa* [20]. These results underscore the strong genomic similarities between these two monocot species, supporting the hypothesis of a more recent common ancestor.

An analysis of the gene structure of *LcBZRs* indicates that the gene structures of most *LcBZR* genes are highly conserved. A conserved motif analysis identified ten motifs in the *LcBZR* genes, all of which consist of the BES1_N domain. This domain is critical for DNA binding, particularly to promoter regions of target genes, thereby regulating downstream gene expression. The BES1_N domain interacts specifically with E-box elements and BR-responsive elements (BRRE motifs), playing a central role in DNA binding, signal transduction, and transcriptional regulation within the BES1 protein family [36,37]. These functions underscore the importance of BES1 in plant growth, development, and stress responses. The structural characteristics of *LcBZR* genes align with findings in other plants such as *A. thaliana* and *O. sativa*, where some members lack introns or contain only a single intron. This pattern has been attributed to intron gain or loss events and the reverse transcription origin of ancestral genes [38]. Consistent with these studies, our analysis revealed that one *LcBZR* gene lacks introns, while the other seven contain only one intron. Given the high homology between *L. chinensis* and *O. sativa*, the intron distribution pattern observed in *LcBZR* genes is likely the result of similar evolutionary mechanisms.

*Cis*-acting regulatory elements are non-coding DNA sequences within gene promoter regions that play a critical role in transcriptional regulation. Their arrangement can provide insights into differences in regulatory mechanisms and functional characteristics among genes [39]. In this study, 15 types of *cis*-acting elements were identified in the *LcBZR* gene promoter regions, grouped into four major categories: hormone response, light response, stress response, and plant development response. The promoter regions of *LcBZR* genes contained diverse *cis*-regulatory elements related to hormone, light, stress, and developmental responses, indicating that the *LcBZR* gene family plays a vital role in integrating multiple signaling pathways to regulate growth and development. Previous studies have shown that BRs, in combination with related hormones such as gibberellins (GA), participate in the regulation of seed germination in *A. thaliana* [40,41]. Other research has indicated that transcription factors of the BZR family play roles in the drought resistance mechanisms of wheat [42]. Additionally, studies have demonstrated that the BZR gene family responds to development-related *cis*-regulatory elements to regulate growth in *A. thaliana* [43]. As shown in Figure 4, multiple transcription factors are likely to interact at these *cis*-acting elements, collectively modulating *LcBZR* gene transcription levels, initiation timing, and expression patterns under different cellular and environmental conditions. This intricate regulatory network highlights the complexity and diversity of transcriptional control mechanisms in the *LcBZR* gene family. Our findings provide valuable insights into the roles of *LcBZR* genes in plant development and their responses to phytohormones and biotic and abiotic stresses.

The BZR gene family demonstrates tissue- and organ-specific expression patterns in various plant species [42]. For example, in *A. thaliana*, BZR genes exhibit relatively high transcriptional activity in roots and buds, while their expression levels are lower in stems, fruits, and flowers [28,44]. In maize, BZR genes are highly expressed in seedlings and endosperm [45]. Similarly, in potato, *StBZR* gene expression varies significantly across tissues, with some genes highly expressed in stems, but weakly expressed in other tissues, and others showing high expression in both petioles and stems [17]. BRs not only are involved in starch utilization, but also play roles in regulating sucrose transport [46]. In sugar beet, BZR genes are pivotal in the accumulation of sugars in the primary root, a process that ultimately promotes increases in root diameter and weight [18]. In our study, *LcBZR* genes were found to have higher expression levels in the roots and leaves of *L. chinensis*. Therefore, it is speculated that these genes may be involved in promoting sugar accumulation, thereby facilitating root expansion. This differential expression among tissues suggests that *LcBZR* genes play vital roles in the growth and development of *L. chinensis*. Furthermore, the observed expression patterns indicate the potential for functional redundancy among *LcBZR* genes, which may contribute to their involvement in multiple biological processes [17,20].

Brassinosteroids (BRs) are essential plant hormones that regulate various aspects of plant growth and development. Through a sophisticated signaling pathway, BRs initiate the dephosphorylation of key transcription factors, BZR1 and BZR2/BES1, which, in turn, modulate gene expression patterns. This intricate regulatory process involves two plasma membrane-localized receptors, BRI1 and BAK1 [47]. When BR levels rise, BRI1 and its co-receptor BAK1 are activated [48,49,50]. Studies have identified BAK1 as a pivotal factor in activating BRI1 kinase, while BSK serves as the substrate for BRI1, enabling downstream BR signaling [51]. Furthermore, it has been suggested that BRI1 and BAK1 cooperatively mediate BSK’s role in BR signal transduction [49,50]. Chen et al. demonstrated that the crosstalk in plant responses and BR signaling primarily depends on upstream components of BZR, such as BAK1 and BSK1 [13]. In this study, simulated grazing treatment of *L. chinensis* revealed that components of the BR signaling pathway, including BAK1, BRI1, and BSK, underwent significant transcriptional changes, showing either upregulation or downregulation. The interaction between BAK1 and BRI1, converging on BSK, indicates that simulated grazing induces substantial alterations in the gene expression of the BR metabolic pathway.

Kim et al. reported that the receptor kinase BRI1 phosphorylates BSK1, facilitating its interaction with the BSU1 phosphatase. BSU1 subsequently dephosphorylates a conserved phosphotyrosine residue (pTyr200), leading to the inactivation of the GSK3 kinase BIN2 [52]. Furthermore, BR signaling suppresses BIN2 activity, triggering the dephosphorylation and accumulation of BZR1, which either directly activates transcription or inhibits the degradation of BZR1 and BZR2/BES1 proteins [47,53]. This study corroborated these findings by demonstrating that in *L. chinensis*, BSK facilitates BSU1’s actions on BIN2, ultimately directing the activation of BZR1/2. The role of BR signaling in transcriptional regulation via BZR has been closely associated with promoting cell elongation, providing a mechanistic explanation for its growth-stimulating effects [54]. In this study, simulated grazing treatment was found to activate the BR signaling pathway in *L. chinensis*, directing BZR1/2 to regulate cell elongation and division. Additionally, Chen et al. emphasized that BZR transcription factors are indispensable for BR signaling and anther cavity development in *Arabidopsis* [13]. Based on these findings, we consider that plant hormone signal transduction plays a crucial role in the mechanism by which *L. chinensis* responds to animal grazing. The mechanism by which animal oral secretions exert their effects may involve regulating the expression of genes within signal transduction pathways, including plant hormone signal transduction, thereby promoting the growth and development of *L. chinensis* and enhancing its defensive capabilities.

## 5. Conclusions

*L. chinensis*, renowned for its exceptional forage quality and resilience to stress, holds a pivotal role in the livestock industry. This study successfully identified eight members of the BZR gene family in *L. chinensis* and provided insights into their potential functions and expression patterns. Furthermore, we examined the alterations in gene expression within the BR signaling pathway under simulated grazing conditions. These findings lay a solid foundation for future investigations into the biological roles of BZR genes in *L. chinensis*. Given that the BZR gene displays significantly higher expression levels in roots compared to stems, leaves, and inflorescences of *L. chinensis*, future investigations may prioritize elucidating the specific regulatory mechanisms governing root expansion. Such research will contribute to a more profound understanding of the functional role that the BZR gene plays in root development in *L. chinensis*.

## Figures and Tables

**Table 1:** Profiles of the BZR gene family members identified in *Leymus chinensis*.

Sequence ID	Gene Name	Number of Amino Acids	Molecular Weight	Theoretical pI	Instability Index	Aliphatic Index	Grand Average of Hydropathicity	Subcellular Localization
Lc2Ns004599.t1	*LcBZR1*	312	33,665.78	8.73	74.56	59.49	−0.599	Nucleus.
Lc3Ns024956.t1	*LcBZR2*	351	37,055.39	8.82	62.31	54.53	−0.507	Nucleus.
Lc7Ns021460.t1	*LcBZR3*	360	37,994.89	7.68	59.62	54.56	−0.608	Nucleus.
Lc2Xm051404.t1	*LcBZR4*	311	33,488.47	7.69	69.15	60	−0.591	Nucleus.
Lc3Xm072826.t1	*LcBZR5*	119	13,939.26	10.18	47.63	75.46	−0.521	Nucleus.
Lc3Xm072827.t1	*LcBZR6*	157	16,553.35	4.88	64.8	54.01	−0.414	Nucleus.
Lc6Xm080063.t1	*LcBZR7*	363	37,694.59	8.72	55.47	52.15	−0.495	Nucleus.
Lc7Xm038258.t1	*LcBZR8*	387	40,634.84	8.14	61.35	53.07	−0.629	Nucleus.

pI: Theoretical isoelectric point.

**Table 2:** Selection pressure analysis of four *LcBZR* gene pairs in *L. chinensis*.

Seq_1	Seq_2	Ka	Ks	Ka/Ks
*LcBZR1*	*LcBZR4*	0.00653678	0.09944666	0.065731521
*LcBZR2*	*LcBZR5*	0.187919356	0.413424421	0.454543433
*LcBZR5*	*LcBZR3*	0.272890959	0.687872785	0.39671719
*LcBZR3*	*LcBZR8*	0.005047338	0.120337557	0.041943162

Ka: nonsynonymous substitution rate; Ks: synonymous substitution rate.

## Data Availability

The raw sequencing data for *L. chinensis* were deposited on NCBI SRA (PRJNA1188712).

## Supplementary Materials

The following supporting information can be downloaded at https://www.mdpi.com/article/10.3390/genes16020155/s1: Table S1: List of *LcBZR* protein sequences; Table S2: The MEME motif sequence and length of *LcBZRs*; and Table S3: *Cis*-acting elements in *LcBZRs*.
